# Substance Use and Psychological Disorders Among Art and Non-art University Students: an Empirical Self-Report Survey

**DOI:** 10.1007/s11469-017-9812-5

**Published:** 2017-09-27

**Authors:** Fruzsina Iszáj, Máté Kapitány-Fövény, Judit Farkas, Gyöngyi Kökönyei, Róbert Urbán, Mark D. Griffiths, Zsolt Demetrovics

**Affiliations:** 1 Department of Clinical Psychology and Addiction, Institute of Psychology, ELTE Eötvös Loránd University, Izabella utca 46, Budapest, 1064 Hungary; 2 Department of Addictology, Faculty of Health Sciences, Semmelweis University, Budapest, Hungary; 3 Nyírő Gyula Hospital, National Institute of Psychiatry and Addictions, Budapest, Hungary; 4 Department of Personality and Clinical Psychology, Institute of Psychology, ELTE Eötvös Loránd University, Budapest, Hungary; 5 Psychology Department, International Gaming Research Unit, Nottingham Trent University, 50 Shakespeare Street, Nottingham, UK

**Keywords:** Art, Psychoactive substance use, Cannabis, Alcohol, Psychological disorders

## Abstract

Media stories often suggest that those working in the creative arts appear to use and abuse psychoactive substances. The aim of the present study was to analyze the relationship between the use of psychoactive substances and the presence of psychological disorders among art and non-art students. Questionnaires related to these two areas were completed by 182 art students in higher education and a control group of 704 non-art university students. To assess psychoactive substance use, a structured questionnaire including the Cannabis Abuse Screening Test (CAST) and the Alcohol Use Disorders Identification Test (AUDIT) was administered to participants. Psychological disorders were assessed using the Hungarian version of the Brief Symptom Inventory (BSI) and the Global Severity Index (GSI). After analyzing the data, significant differences were found between the two groups regarding their first use of psychoactive substances. Art students’ current substance use was found to be significantly more frequent compared to the control group. In relation to psychological disorders, art students scored significantly higher on three scales of the BSI (i.e., psychoticism, hostility, and phobic anxiety). Overall, a significantly higher proportion of artists were labeled as “problematic” using the GSI. The results suggest that artists have a higher risk of both substance use and experiencing psychological disorders.

## Artists’ Substance Use and the Introduction of the “Balancing” Phenomenon

Stories in the media often suggest that those working in the creative arts appear to use and abuse psychoactive substances (Iszaj et al. 2016). Knafo (2008) identified possible reasons for artists’ substance use as an aid to depersonalization and derealization leading to the estrangement of personality and reality experiences and the achievement of heightened and altered perceptual states. Such phenomena are also observable in psychotic individuals, but many individuals intentionally search for mind-altering experiences using techniques such as practicing meditation or yoga. Another reason that artists may engage in psychoactive substance use is to see the world around them from a different perspective. Knafo (2008) defined this intention as the regressive reliving of earlier self-states and object relations, and the wish to stimulate unusual modes of cognition. The same author also contended that isolation and insecurity are necessary elements of artists’ lives that are useful in handling the stress of social situations that they find difficult to tolerate. Given such circumstances, substance use provides psychological support to artists. Feist (1998) asserts that highly creative people need solitude for creation. However, the feeling of isolation can be both inspiring and threatening (Kohut 1971).

Ehrenzweig (1970) claims “the hidden order of art” is an ability that most adults lack but many artists retain because of their *oversensitivity*. Here, the state in which knowledge, feelings, and cognitive and affective processes is not yet differentiated as childlike and regressive. If artists feel that this ability might be lost, chemical substances might be used to facilitate the desired regressive state. Freud (1955) compared the creative act to childish play. Both artists and children create their own reality which they take very seriously. Consequently, it is perhaps understandable that borderline personality disorder (Kernberg and Michels 2009) is strongly connected with both substance use (Trull et al. 2000; Verheul 2001) and creative work. A possible reason may be that borderline functioning might be able to facilitate the creative work via impulsivity, emotional instability, and a temporarily slackened relationship with reality.

At the same time, artists may experience increased drug use as a problem in relation to the balancing of the instability. In the *inspirational phase* of the artistic creative process (Kris 1962), substances may help in disinhibiting the cognitive blockades and complexes as well as occasionally providing artists with a childlike way of thinking. When using psychoactive substances, artists may be able to contact deeper levels of their psyche more easily. At the same time, during artistic creation, substances may, on the contrary, reduce emerging anxiety and distress as potential increments of the work with the unconscious, even if artists do not use substances for creative work at all. In this respect, the role of psychoactive substances, such as alcohol, benzodiazepines, and opiates, which can have depressant effects, may be emphasized. In addition to regression seeking, another reason for artists’ substance use might be the reduction of the anxiety, which can be experienced as the result of regression. In one study, the “balancing” role of opium was hypothesized in two literary figures’ case—Edgar Allan Poe and Samuel Taylor Coleridge—where opium had a role in the emotional regulation of both writers (Iszáj and Demetrovics 2011).

Another cause of using psychoactive substances might be the redirection of the libido and the influence of personality through reordering the soul’s powers which might mean both the broadening and narrowing of consciousness (Dobkin de Rios and Janiger 2003). Many famous substance user examples are present among creative artists, such as Tennessee Williams who drank alcohol heavily and took sleeping pills from the age of 25 years. He also used stimulants frequently for work. Later, in his fifties, amphetamines were prescribed for him—which he got addicted to—as well as barbiturates (Jeste et al. 2004). Alcoholism is strongly connected not only to literary figures (Tolson and Cuyjet 2007), but heavy alcohol use is very common among jazz musicians too (e.g., Charlie Parker or Billie Holiday). Preti and Vellante (2007) reported in a study of 80 professional artists and 80 controls, significantly more psychoactive substances were used by artists. In case of legal substances, hardly any difference was found, but artists used illegal drugs significantly more compared to the control group. Andreasen (1987) reported a higher rate of alcoholism among creative writers (30%) compared to control participants (7%).

### Artists’ Enhanced Sensitivity and Psychological Disorders

During the artistic creative act, artists use both conscious and unconscious processes, suggesting that they possess heightened sensitivity (Knafo 2008). This implies a greater ability to use and react to emotions, and a higher tolerance of extreme emotional conditions. Artists observe things that less sensitive people might not. However, being highly sensitive might mean greater risk of developing mental illnesses. Artists tend to be more pathological than less creative individuals (Knafo 2008). Eysenck (1993) found that psychotic and creative individuals are both characterized by wide associative horizons and overinclusive thinking that might lead to remote associations (i.e., creative inspiration).

Jung (1984) theorized that if artists want to reach the freedom of expression, they have to get into the state of “passive sensibility”. During the acceptance of such emotional submissions, artists sustain the possibility of losing their ego, reality, control, and contingent reactivation of traumas. Working with the unconscious conceals risks, and artists might face emotions and impulsions that are difficult to handle. Jung (1984) further emphasized that during artistic creation, the shrinking of consciousness may lead to great psychic suffering. Additionally, artists appear to be close to collective unconscious, going beyond their personal experiences, and feelings, experiencing a certain amount of relief. Creative individuals have a duality in their lives. On the one hand, they have personal lives, and on the other, they are impersonal, creative beings. Because of this duality, their lives are potentially full of psychological struggles (Jung 1971).

Andreasen (1987) reported a surprising number of suicides committed by writers in the twentieth century (Sylvia Plath, John Berryman, Virginia Woolf, etc.). Furthermore, the incidence of bipolar disorder is more frequent in creative writers and their relatives than in any other population (Gardner 1997). Several case studies connected to artists’ psychological disorders are present in the literature. Rihmer et al. (2006) wrote that affective disorders were common among the family of Robert Schumann. He had several manic and depressive phases and he tried to commit suicide twice, unsuccessfully. Tennessee Williams suffered from lifelong bipolar disorder (Jeste et al. 2004). In Ernest Hemingway’s family, five suicides were committed within four generations (Roy et al. 1997). Rihmer et al. (2006) noted that Hemingway also had bipolar disorder.

Despite such high-profile examples, very few examples can be found in the empirical literature. Preti and Vellante (2007) conducted a study, in which 80 professional Italian artists were compared to 80 controls. Artists were reported to have more unusual delusion-like experiences scoring higher on Peters et al. Delusions Inventory (PDI; Peters et al. 1999) that might support the association between schizotypy scores and creativity. However, the authors added that artists’ higher rate of substance use might explain the higher scores on PDI. Andreasen (1987) studied 30 creative writers who were compared to 30 controls. The results of the structured interviews refuted the notion that creativity was strongly associated with schizophrenia. Eighty percent of the writers had had a period with affective illness(es) in their lives, while only 30% with the same disorder was observed among controls.

Apart from clinical descriptions and case studies, very few studies have investigated psychoactive substance use and psychological disorders among artist populations. Consequently, the aim of the present study was twofold. The first was to compare the legal and illegal substance use of students in art and non-art higher education. Secondly, to examine whether different psychological symptoms have different roles regarding substance use in these two samples.

## Methods

### Samples

#### Artist Sample

The artist sample comprised of students in higher education in the field of arts. Data were collected from three Hungarian universities of fine arts and design. Altogether, 130 art students participated in the study. The sample comprised 26.2% males. The mean age was 22.06 years (SD = 2.09 years).

#### Non-artist Sample

As a comparison (control) group, 698 university students of non-art studies participated and comprising 42.4% males. The mean age was 23.8 years (SD = 1.33 years). Participant recruitment took place simultaneously in arts and non-art faculties. The numbers of participants in each group simply reflect the data collected in each cohort via convenience sampling.

All participants provided informed consent. Anonymity was assured in all cases. Respondents completed the questionnaires individually and returned them to the researchers in sealed envelopes. The study was approved by the Institutional Review Board of the ELTE Eötvös Loránd University.

### Measures

#### Psychoactive Substance Use

To assess the participants’ psychoactive substance use, a structured questionnaire was used. The questionnaire contained items regarding the use of several legal and illegal psychoactive substances, including tobacco, alcohol, cannabis, ecstasy (MDMA), amphetamines, cocaine/crack, heroin (and other opiates), LSD, psychoactive mushrooms, GHB, solvents, combination of alcohol benzodiazepines, and benzodiazepine use without prescription. The age of the first use of psychoactive substances was also assessed. In the case of tobacco, both the age of the first experimenting and the starting age of regular smoking was assessed. Similarly, in the case of alcohol, both the first age of alcohol use and getting drunk for the first time was asked. Additionally, the frequency of past month and past year alcohol and cannabis use was also assessed. As a brief screening method for excessive drinking, we used the Hungarian version of the Alcohol Use Disorder Identification Test (AUDIT) (Babor et al. 2001; Gerevich et al. 2006). For the assessment of cannabis use and related problems, the Hungarian version of the Cannabis Abuse Screening Test (CAST) (Legleye et al. 2007; Gyepesi et al. 2014) was applied.

#### Psychological Disorders

The Brief Symptom Inventory (BSI) containing 53 items was completed by all participants (Urbán et al. 2014, 2016). The BSI is one of the most widely used self-report tests for assessing psychological problems. Participants rated each item on a 5-point scale ranging from 0 (not at all) to 4 (extremely) measuring psychological distress in the past seven days. The Global Severity Index (GSI) was also administered (Derogatis and Melisaratos 1983) in order to assess overall psychological distress level. It is calculated by taking the mean of all subscale scores.

### Statistical Analysis

Since the groups of artists and non-artists significantly differed in both age (*t* = −12.35, *p* < 0.001) and gender distribution (χ2 = 12.08, *p* < 0.01), these potentially confounding variables were controlled for. Age and gender (1 = male, 2 = female) were used as covariates in a series of regression models in which the exogenous variable was the grouping variable of being an artist (value of 0) or non-artist (value of 1), and endogenous (dependent) variables were the number of the types of substances ever used, the age of the first use of cannabis, the age of the first drunkenness, BSI factors, Global Severity Index, and past month frequency of alcohol and cannabis use. Models were analyzed using Mplus 6.0 software (Muthén & Muthén, 1998–2007).

The internal consistencies of the nine symptom scales were calculated using SPSS 17 somatization (SOM) Cronbach’s α = 0.79; obsessive-compulsive (O-C) Cronbach’s α = 0.73.; interpersonal sensitivity (I-S) Cronbach’s α = 0.71; depression (DEP) Cronbach’s α = 0.83; anxiety (ANX) Cronbach’s α = 0.73; hostility (HOS) Cronbach’s α = 0.76; phobic anxiety (PHOB) Cronbach’s α = 0.67; paranoid ideation (PAR) Cronbach’s α = 0.68; psychoticism (PSY) Cronbach’s α = 0.66.

Past year and past month frequencies of cannabis and alcohol use among artists and non-artists were compared by using chi-square tests. Finally, a path analysis was conducted using Mplus software in order to explore whether the grouping variable (artist vs. non-artist) on past month frequency of alcohol and cannabis use was mediated by the severity of psychiatric symptoms (GSI). An MLR estimation (maximum likelihood estimation with robust standard errors) was used in all of these models. Acceptability of the models was based on goodness of fit indices. A model is acceptable if root-mean-square error of approximation (RMSEA) < 0.08, comparative fit index (CFI) > 0.95, non-normed fit index or Tucker-Lewis index (TLI) > 0.95. However, all of the models were saturated ones without degrees of freedom; thus, fit indices had no relevance in these cases (RMSEA = 0.000; CFI = 1; TLI = 1).

## Results

**Fig. 1:**
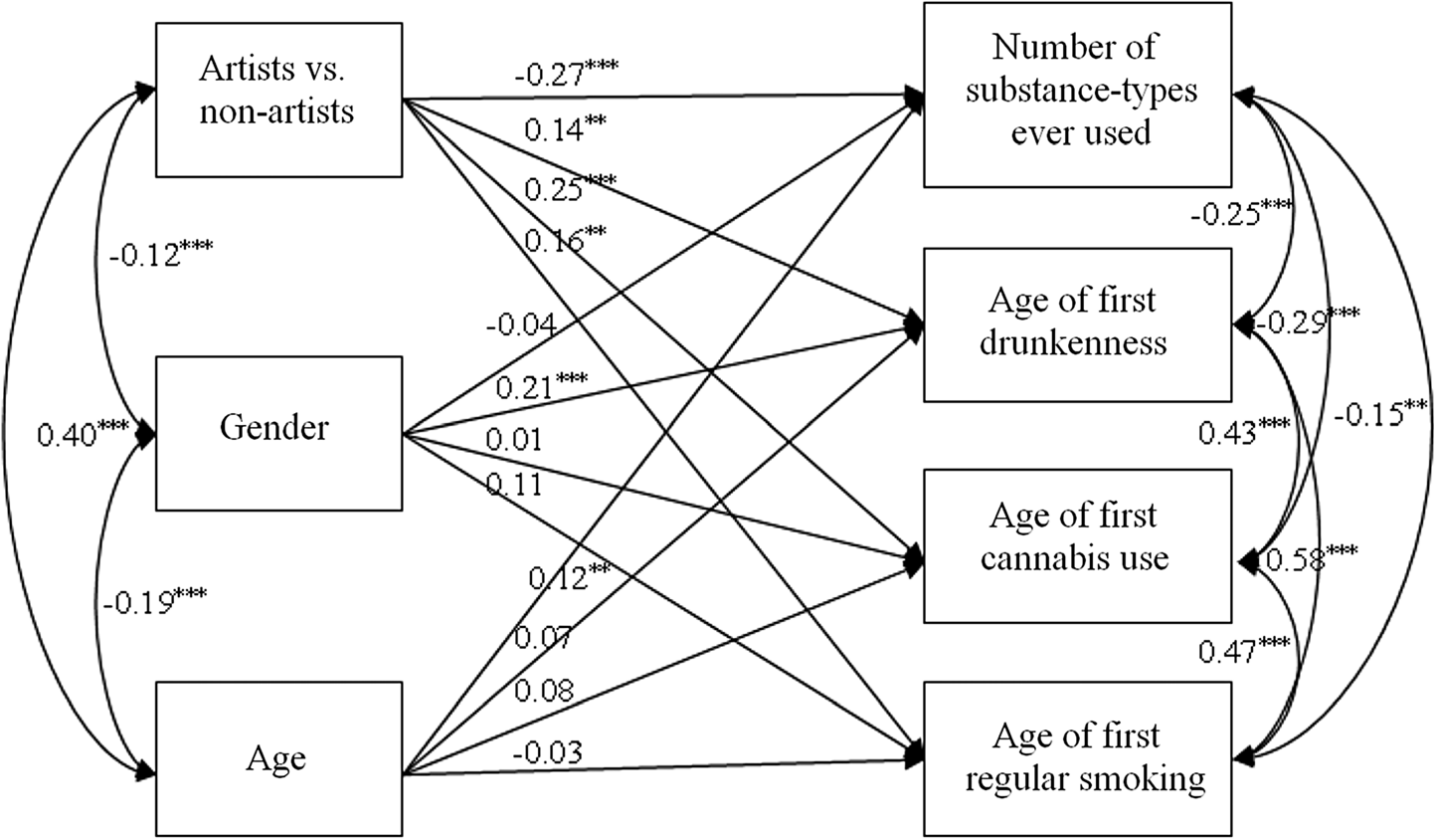
Association between being an artist and specific aspects of substance use (onset of use, types of substances ever used) (*p < 0.05; **p < 0.01; ***p < 0.001)

In the first regression model, the number of types of psychoactive substances ever used and the age of the first substance use regarding the listed substances were endogenous variables. The grouping variable of artists vs. non-artists was the exogenous variable, and age and gender were covariates (Fig. 1). Out of the listed psychoactive substances, only the age of the first cannabis use, the age of the first drunkenness, and the age of the first regular smoking in the model was used since significant differences were only found in these cases (age of first cannabis use *t* = −5.81, *p* < 0.001; age of first drunkenness *t* = −3.78, *p* < 0.001; age of first regular smoking *t* = −2.31, *p* < 0.05) between artists (age of first cannabis use 16.70, SD = 2.12; age of first drunkenness 15.56, SD = 2.03; age of first regular smoking 16.96, SD = 2.15) and non-artists (age of first cannabis use 18.36, SD = 2.13; age of first drunkenness 16.48, SD = 2.25; age of first regular smoking 17.77, SD = 2.27).

The grouping variable of being an artist or non-artist had a significant predictive value on all the dependent variables, the number of types of substances ever used (ß = −0.27, *p* < 0.001), the age of the first drunkenness (ß = 0.14, *p* < 0.01), the age of the first cannabis use (ß = 0.25, *p* < 0.001), and the age of first regular smoking (ß = 0.16, *p* < 0.01). Regarding the covariates in the model, age had a significant predictive value on the number of types of substances ever used (ß = 0.12, *p* < 0.01), but neither on the age of the first drunkenness (ß = 0.07, *p* > 0.05), nor on the age of the first cannabis use (ß = 0.08, *p* > 0.05), or on the age of first regular smoking (ß = −0.03, *p* > 0.05). Gender as the other covariate had a significant predictive value on the age of the first drunkenness (ß = 0.21, *p* < 0.001), but neither on the number of types of substances ever used (ß = −0.04, *p* > 0.05), nor on the age of the first cannabis use (ß = 0.01, *p* > 0.05), or on the onset of regular smoking (ß = 0.11, *p* > 0.05).

Analyzing the past year and past month frequency of alcohol and cannabis use, significant differences were found. In order to meet the assumption of expected cell count (five or more in all cells) regarding the chi-square test, the five response categories of past year and past month cannabis and alcohol use were merged into three categories in cases of past year frequency of cannabis and alcohol use, and past month frequency of alcohol use, whereas in case of past month frequency of cannabis use only two categories were used. While 29.0% of artists used cannabis on a weekly basis in the past year and 19.0% stated the same for the past month, this was only true for 10.7% and 7.4% of the controls. Similarly, while 8.5% of the artists drank alcohol more than 20 times during the past month this was only true for 3.7% of the comparison group (Table 1).

**Table 1:** Differences in the past month prevalence of alcohol use and the past month and past year prevalence of cannabis use among the two groups

Cannabis		Artists	Non-artists	χ^2^	Effect size *r*
Last year prevalence *N* (%)	0 times	19 (30.6%)	30 (18.9%)	18.79***	*r* = 0.29
	Monthly	25 (40.3%)	112 (70.4%)		
	Weekly or more	18 (29.0%)	17 (10.7%)		
Last month prevalence *N* (%)	< 3 times	51 (81.0%)	150 (92.6%)	6.45*	*r* = 0.17
	Weekly or more	12 (19.0%)	12 (7.4%)		
Alcohol		Artists	Non-artists	χ^2^	
Last year prevalence *N* (%)	Monthly or less often	40 (31.0%)	264 (37.9%)	5.62	*r* = 0.08
	2–4 times a month	53 (41.1%)	299 (43.0%)		
	Weekly twice or more often	36 (27.9%)	133 (19.1%)		
Last month prevalence *N* (%)	< 3 times	64 (49.6%)	410 (58.9%)	7.79*	*r* = 0.09
	4–19 times	54 (41.9%)	260 (37.4%)		
	> 20 times	11 (8.5%)	26 (3.7%)		

*p < 0.05; **p < 0.01; ***p < 0.00

With regard to the psychiatric symptoms (using the BSI), the grouping variable of artists vs. non-artists was a significant predictor of higher mean scale score in cases of three of the nine BSI scales: psychoticism (ß = −0.10, *p* < 0.05); hostility (ß = −0.11, *p* < 0.05) and phobic anxiety (ß = −0.18, *p* < 0.001). Gender as a covariate had a significant predictive value on the mean scale score of somatization (ß = 0.12, *p* < 0.01), obsessive-compulsive (ß = 0.08, *p* < 0.05) and anxiety (ß = 0.11, *p* < 0.01). Age as the other covariate had no significant predictive value on any of the BSI scales’ mean scores. When the Global Severity Index (GSI) was used as the endogenous variable, only the grouping variable (artists vs. non-artists) had a significant predictive value on GSI score (ß = −0.08, *p* < 0.05). The covariates, age (ß = −0.01, *p* > 0.05) and gender (ß = 0.06, *p* > 0.05) did not.

### SEM Path Analysis

**Fig. 2:**
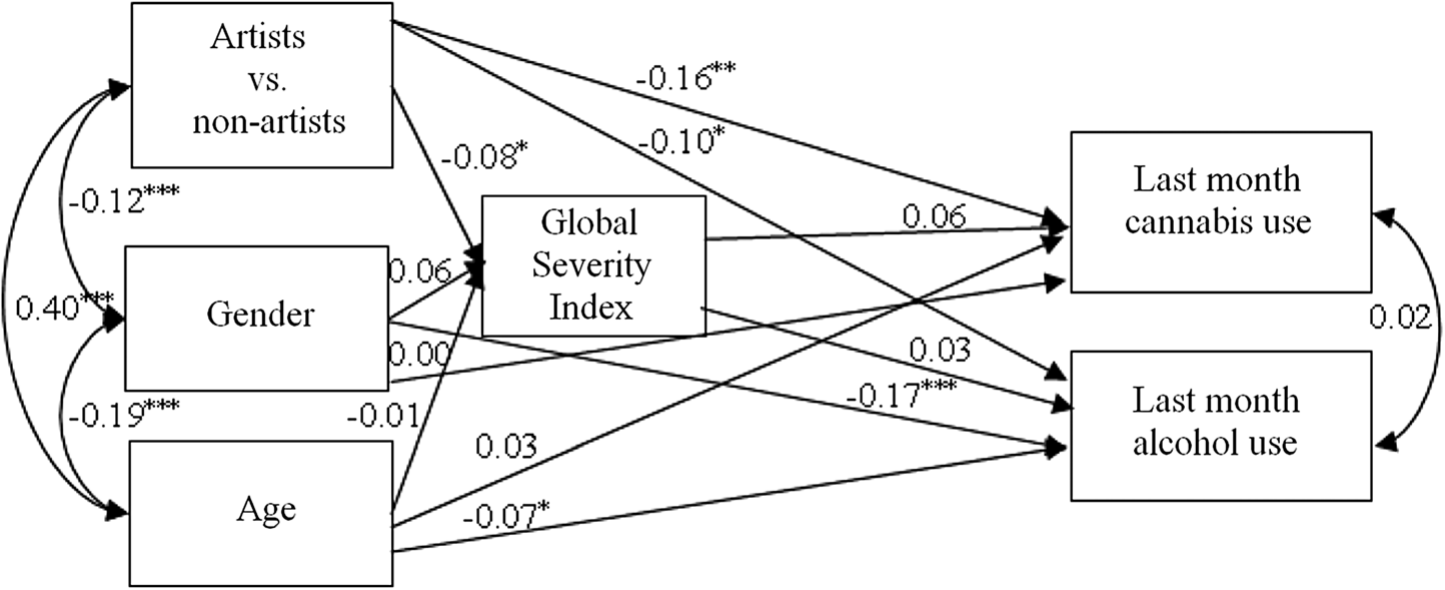
Path analysis model of past month alcohol and cannabis use (*p < 0.05; **p < 0.01; ***p < 0.001)

Based on the results, a path analysis was conducted in order to explore whether or not the severity of psychiatric symptoms (GSI) as endogenous variables had a mediating effect between the grouping variable of being an artist or non-artist, or age and gender as covariates, and the frequency of past month alcohol use and past month cannabis use (0 = no alcohol or cannabis consumption, 1.5 = on 1–3 occasions, 6.5 = on 4–9 occasions, 14.5 = on 10–19 occasions, 25 = on 20 occasions or more, 30 = on every day) as endogenous (dependent) variables (Fig. 2).

The grouping variable of being an artist or non-artist had significant predictive value on the frequency of past month alcohol (ß = −0.10, *p* < 0.05) and cannabis use (ß = −0.16, *p* < 0.01), as well as on GSI’s mean score (ß = −0.08, *p* < 0.05). Age and gender as the covariates had significant predictive value on the frequency of past month alcohol use (age ß = −0.07, *p* < 0.05; gender ß = −0.17, *p* < 0.001), but had no significant predictive value on the frequency of past month cannabis use (age ß = 0.03, *p* > 0.05; gender ß = 0.00, *p* > 0.05) or on GSI’s mean score (age ß = −0.01, *p* > 0.05; gender ß = 0.06, *p* > 0.05). GSI as the mediator had no significant predictive value neither on the frequency of past month alcohol use (ß = 0.03, *p* > 0.05), nor on the frequency of past month cannabis use (ß = 0.06, *p* > 0.05).

## Discussion

In the present study, art and non-art university students’ use of psychoactive substances and possible mental disorders was examined. The results showed that being an art student might be a risk factor concerning an early onset of substance use (first drunkenness; first cannabis use; first regular cigarette smoking). Given that this was a cross-sectional study, it may also be the case that using psychoactive substances earlier in their lives may be a factor in becoming an art student rather than a non-art student. Given that psychoactive substance use may affect academic performance (e.g., Lynskey and Hall 2000; Macleod et al. 2004; Murray et al. 2012), it could be the case that grades for non-art subjects (such as mathematics and science) are affected more by substance use/abuse than for art subjects, and that the choice of higher education degree was a function of grades attained during earlier psychoactive substance use rather than art being a risk factor for early psychoactive substance use.

Art students also tried more types of substances compared to the control group. Again, engaging in greater psychoactive substance use might have led to poorer grades and increasing the chances of taking art-based subjects at university. Furthermore, more frequent alcohol and cannabis use was observed among art students with regard to current substance use. In addition, art students scored significantly higher on three of the nine BSI scales. Given that increased psychoactive substance use is often accompanied by other psychological comorbidities (e.g., Degenhardt et al. 2001; Neighbors et al. 1992; Newbury-Birch et al. 2002) it is perhaps unsurprising that there were significant differences on some of the BSI subscales. However, the GSI as a mediator did not show a significant relationship with the frequency of psychoactive substance use. This may have been because GSI scores are calculated as a mean of all the subscales and only a few behaviors on both the GSI and BSI subscales demonstrated significant differences.

The finding that art students tended to use substances more frequently strengthens the arguments of Preti and Vellante (2007). However, in their study, artists tended used more illegal psychoactive substances. In the present study, no such evidence was found (although this may have been due to the relatively small number of art students compared to the number of non-art students in the sample). More specifically, both alcohol and cannabis were more significantly consumed by artists as compared to non-art students. Therefore, the balancing effect of psychoactive substances can be assumed because the population of art students tended to balance their extreme emotional conditions that are present during artistic creation as assessed by their psychiatric symptoms in this study.

Art students were found to have more severe psychiatric symptoms and such a finding appears to confirm the theoretical considerations related to artists’ enhanced sensitivity (Knafo 2008) and the assumption that they are more likely to develop mental disorders (e.g., Andreasen 1987). However, no data exist outlining any primary mental disorder of the population sampled in the present study. In relation to gender, the first drunkenness of females occurred later than in the case of males. Moreover, a significant correlation was found between the age of first drunkenness and first cannabis use. Both of these variables also showed a significant correlation with the age of first regular cigarette smoking. This chain of experimenting with substances at an early age may be associated with regression seeking as reported in earlier case studies of two literary figures (i.e., Edgar Allan Poe and Samuel Taylor Coleridge; Iszáj and Demetrovics 2011). However, it is also worth noting that age of first drunkenness has been shown to be a more important risk factor in later psychopathology than age of first drinking (e.g., Kuntsche et al. 2013) and that early alcohol abuse may have also affected education and the choice of degree in higher education.

The study is not without its limitations. The sample was self-selecting and not necessarily representative, and all the data were self-report and subject to well-known biases (social desirability, recall biases, etc.). Furthermore, all the participants were Hungarian and may not be generalizable to other populations. Despite these limitations, it might be concluded, as hypothesized, that artists showed higher frequencies of substance use as well as a greater possibility of developing mental disorders. However, the possible association between substance use and psychological disorders was not confirmed. Due to the quantitative and cross-sectional nature of the study, the underlying causes of these mechanisms are not clear. Longitudinal research would be needed to confirm the hypotheses and speculations made in the present paper. Earlier in the paper, several possible causes for substance use among artists were introduced. Whether enhanced substance use is the result of one of these causes or not, remains an open question. In this respect, future research should also focus on qualitative aspects. Related to this complex issue, the question arises whether substance use in general is only a result of the individual’s personality traits and/or is it specific to artists and connected to emotional regulation or whether there are other factors that are involved in this relationship.

Findings in the present study clearly show that artists presented with psychological disorders at a higher rate than the non-artist control group. The extension or alteration of the sample could be an interesting further step in research. For instance, changing or adding other age groups, or examining the topics in a more nuanced way (e.g., examining particular types of artist or particular types of degree subject), the development of these two phenomena might be better captured. This could contribute to the better understanding of the long-term causes and effects of artists’ psychoactive substance abuse and psychological disorders. Another research objective might be the inclusion of other non-Hungarian samples to examine cultural diversity. By studying art vs. non-art students of other countries, a wider and more nuanced understanding of the relationship between these phenomena may be reached.
